# Is Corporate Social Responsibility Considered a Marketing Tool? Case Study from Customers’ Point of View in the Slovak Food Market

**DOI:** 10.3390/foods12142770

**Published:** 2023-07-20

**Authors:** Kristína Igarová, Zdenka Kádeková, Ingrida Košičiarová, Milan Džupina, Marek Dvořák, Luboš Smutka

**Affiliations:** 1 Faculty of Economics and Management, Institute of Marketing, Trade and Social Studies, Slovak University of Agriculture in Nitra, 94976 Nitra, Slovakia; xigarova@uniag.sk; 2 Department of Mass Media Communication and Advertising, Faculty of Philosophy, Constantine the Philosopher University in Nitra, 94901 Nitra, Slovakia; mdzupina@ukf.sk; 3 Department of Trade and Finance, Faculty of Economics and Management, Czech University of Life Sciences Prague, 16500 Prague, Czech Republic; dvorakmarek@pef.czu.cz (M.D.); smutka@pef.czu.cz (L.S.)

**Keywords:** consumer, perception, food marketing, awareness, rational consumer behavior, irrational consumer behavior, food market, CSR

## Abstract

The paper aims at the question of using Corporate Social Responsibility (CSR) activities as a marketing tool from the customers’ perspective in the Slovak food market. Five research questions and hypotheses were set to reach the given aim. The research was based on a questionnaire survey with 1254 respondents. The frequency and contingency tables were used to evaluate the obtained data, one sample proportion Z test, Pearson Chi-square test, and Spearman’s rank correlation coefficient. Based on the results, although the customers are familiar with CSR, many respondents need to learn more about these activities. CSR activities must be more actively applied and communicated by the food companies. In most cases, Slovak customers perceive the sustainability of food companies and their CSR activities as a marketing communication tool that can build a positive image of the company. At the threshold of the 3rd millennium, CSR is also connected with rationality and irrationality in creating preferences in consumer shopping behavior. While the way that food is produced can be changed through regulation, the communication of CSR and sustainability activities are major drivers for the development of food companies. Trustworthiness is one of the key factors, and customers play a key role in this direction. The solved issue has a huge impact on the success of the food companies in the market, and, therefore, it would be suitable to pay attention to this issue and conduct similar research in other E.U. countries and on their food companies.

## 1. Introduction

Currently, a popular topic is Corporate Social Responsibility (CSR). To behave socially responsibly is a must. Food and beverage market companies are increasingly interested in building reputation and brand awareness through socially responsible activities. Therefore, the basis for proper functioning in this area can be setting goals and target groups that they will focus on in their CSR program. The public very sensitively observes the CSR activities of each company on the market. There is a direct link between CSR and company performance. Although these activities are a large and irreversible investment in the short term, the opposite is true. In the long term, these investments will return to the company; satisfaction is not mainly customer loyalty. Therefore, they perceive the organization and the public as interconnected. The way to correctly use CSR activities in public relations (P.R.) is to use publicity. The basis of P.R. is influencing public opinion. Publicity as a P.R. tool is how organizations convey information to the media, and it is up to them to decide whether to publish this information about the company. If this information is published, it gains much more interest and trust from the general public than the advertisement itself, which is perceived subjectively. The possibilities of publications range from interviews, reports, and email marketing, via the Internet, and lectures to many other printed tools. Establishing relations with journalists to spread a brand or company’s good name and inform the public about socially responsible activities practiced by companies is very challenging. Frequently, much effort goes into the company.

Most food companies already act socially responsibly and show various activities; however, customers cannot connect with the concept of CSR or consider it only as a marketing strategy. As food companies try to build the company’s good name and brand image with these activities, the public can view these activities as untrustworthy. CSR activities in marketing communication can greatly benefit companies, increasing the sale of goods and services and creating competition in the food market. Interest within our study was aimed at trustworthiness and is one of the key factors in this direction for customers and their consumer behavior, which the CSR activities in the food market can influence. 

Our study was organized based on the necessity to fill out the gap in this area of research. It is still needed to carry out research with such a focus in Slovakia. 

**Figure 1:**
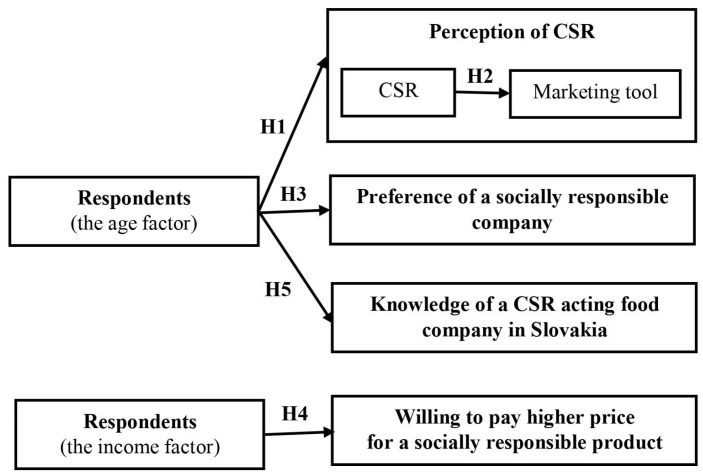
The theoretical model of our research. Source: Authors’ elaboration.

Concerning CSR and the food market in the Slovak Republic, professionals from the marketing, management, and economy conducted research based on the questionnaire survey focused on CSR activities as a marketing tool in the food market from the customers’ perspective. Firstly, the most important problems and issues related to the topic were investigated and transferred to the online questionnaire. We ensured the representativeness of the research sample by addressing the questionnaire to the target interest groups and clubs focused on the food market, including respondents of all ages. It is always useful to visualize the research in the form of a model [1,2,3], which allows us to identify the underlying relationships better. The theoretical model of our research was as follows (Figure 1).

The survey results with 1253 participating respondents showed that, although they are familiar with the concept of CSR, many respondents either need to learn these activities or pay more attention to them. This may be because some CSR activities need to be actively applied and communicated by food companies.

Based on our conducted research, customers perceive the CSR activities of food companies in most cases as a marketing communication tool that builds the company’s good name and image. We can also observe a huge influence of rationality and irrationality in creating preferences in consumer shopping behavior based on CSR activities and their implication and communication with customers. The submitted contribution creates a solid basis for further research and practical application in using CSR activities in Slovakia and E.U. countries on the food market. It can be used as a base for further research in this area.

### Literature Review

The food market is very competitive; thus, it is challenging to attract and retain customers in the food sector of the economy [4,5]. The agricultural and food sectors are key to food security [6]. Although the government is the subject that is mainly responsible for social issues, progressive companies take advantage of differentiation from their competitors and pay more attention by implementing CSR plans and practices [7]. Since the end of the 20th century, the philosophy of CSR—socially responsible entrepreneurship in which companies decide to implement in their business strategies and activities decisions that contribute to a better state of society and economically sustainable growth [8]—is increasingly gaining awareness and interest among companies. Usually, we tend to think of environmental issues when talking about CSR. However, CSR, as a concept based on sustainability, also deals with other social and economic aspects of sustainability. Corporate Social Responsibility (CSR) can be viewed as a set of responsible practices and behaviors that shift organizations towards more responsible behavior [9] and improve the quality of life of the local community. CSR has also become a central theme in the food industry, mainly regarding sustainability [10]. Other reasons exist for implementing CSR practices in the food industry [11,12]. Overall, the CSR strategy has a significant potential to create a sustainable competitive advantage [11,13] and help to expand to other countries with different cultures [14]. From a consumer point of view, CSR implementation in corporate strategies has many advantages. CSR activities improve both corporate and brand image, which can satisfy the external audience [15]. Subsequently, it can increase the stock value [16].

Moreover, it can positively affect customer loyalty and influence the decision-making process. It mainly applies when more similar or identical products are on the market. The assumption is that customers will choose the one with the socially responsible image–conscious customers [17,18,19].

The food sector is dominantly focused on influencing the number of conscious customers who care about CSR issues [20]. As for the agri-food sector, the food companies are often referred to as responsible for (i) negative environmental impacts and depleting natural resources and biodiversity [21], (ii) implementing safety standards affecting people’s health and life [22], and (iii) applying unfair trading practices due to the considerable bargaining power in the supply chain. The solution for the negative image of the food brands might be to apply responsible marketing practices based on presenting CSR through marketing tools. It led many companies to develop comprehensive CSR strategies integrated into marketing [22]. Currently, food companies heavily invest in responsible marketing [23]. Implementing CSR in marketing communication is, of course, not a completely new phenomenon. Companies adopted CSR content to highlight responsibility and sustainability issues in their communication strategy [24,25]. The companies are motivated to penetrate international trade using CSR philosophy and strategy and build the positioning strategy on the domestic market. The reason is that public opinion is more conscious about global issues as the environment is becoming an important social responsibility pillar [26]. Still, there are slight differences between CSR and responsible marketing. The key difference is that responsible marketing aims to help businesses by determining a target market’s needs, wants, and interests more effectively and efficiently than competitors in a way that preserves or enhances the well-being of the individual consumer and society in general [27]. Implementing it correctly can improve the company’s image or increase market share. It has to be used for the right purposes, or it can harm the company’s image or goodwill [28]. Responsible marketing (societal marketing) should not be confused with social marketing [29], which usually focuses on changing behavior to improve public health and ensure environmental protection or social well-being. Societal marketing is becoming an increasingly important concept in the food sector as customers closely watch the steps of companies. For CSR strategies, it is important not only to bother about production and sales but also to communicate such actions to the audience. Thus, through donations, sponsorship, foundations, endowment funds, and reporting, socially responsible businesses could be viewed as marketing tools [30]. The right and balanced communication strategy is key to generating positive associations and preventing negative attitudes toward the company [31].

As mentioned above, consumers become more conscious about responsibility issues. Consumer expectations related to social responsibility have increased over the last years as the number of companies with CSR projects has grown. Consumers expect companies to engage in social responsibility and communicate their corporate social responsibility (CSR) efforts to a varied, influential, alert audience [32]. Also, the number of companies that actively communicate their CSR practices has increased. Organizations adopt CSR to strategically “integrate economic, social and environmental concerns into their business strategies, management tools, and activities, going beyond compliance and investing more into human, social and environmental capital” [33].

Regarding consumer behavior, the consumers’ concern about CSR pushes companies to recognize their obligations toward society and study and mitigate their environmental impacts [34]. The shift in consumer behavior can be explained by the social exchange theory, which states that social behavior results from exchange processes to maximize benefits or minimize costs between parties (a company and a consumer) [35]. The consumers acquire certain cognitive beliefs about the business, evaluate them, and are led to behavioral manifestations concerning the business. Conscious consumers support businesses that trigger positive social change and include CSR criteria in their criteria influencing consumer behavior [36]. In other words, they try to avoid companies that do not care about society. Moreover, they actively seek out products from businesses that help society. CSR is important for attracting new and existing customers, as they help new customers choose a product or firm [37].

## 2. Methodology, Materials and Methods

This research investigates awareness of CSR practices across different generations in the food industry and its implications for consumer decision-making. Here is also observed an influence of rationality and irrationality in creating preferences in consumer shopping behavior based on CSR activities in the food market.

Several studies try to find the relationship between CSR and customer satisfaction and loyalty [38,39,40,41]. The study contributes to existing studies on CSR practices in the food industry, providing new information on the range of socially responsible activities commonly used in the food market. Engizek and Yasin [42] highlighted in their research that there is a need to investigate CSR attitudinal and behavioral customer outcomes. Companies’ activities towards applying CSR in their practice are intensively perceived and assessed by the internal and external public. They represent an important marketing tool, especially in communication policy. By their very nature, food companies and the food market are the closest to the urgency of applying the CSR concept. CSR is currently an important key aspect of the success of companies.

For this reason, we decided to conduct research in Slovakia and find out the current situation in the food market and the application of CSR activities in food companies. We are aware that, currently, this phenomenon can strongly influence consumer behavior in particular.

### 2.1. Sampling and Data Collection

In order to find out and collect the necessary data, we decided to conduct primary research. An online questionnaire was developed and submitted to consumers from different generations to verify their knowledge about Corporate Social Responsibility, their perception of CSR, and their attitudes to CSR implemented by the food sector. The questionnaire was distributed via social media platforms and email. We ensured the representativeness of the research sample by addressing the questionnaire to target interest groups, including respondents of all age categories.

In order to fulfill the stated goal of the presented research paper, we have chosen a suitable form of data collection when involving 1253 respondents from Slovakia, 602 women and 652 men. In contemplation of collecting the necessary data and ensuring the sample’s representativeness in terms of age, we distributed the questionnaire among various interest groups, such as student and retirement groups, to include each age category.

### 2.2. Definition of Research Questions and Hypotheses

The main goal of the research was to determine whether food companies’ CSR is considered a marketing tool. In the presented research, we assumed that the results would provide us with answers to various questions about the given issue. We were interested in whether respondents know and are familiar with the concept of CSR and whether they know specific food companies based in Slovakia that apply and are dedicated to CSR. Furthermore, we were aware of whether the CSR of food companies affects consumer behavior and, if so, which age groups.

When formulating the research questions, we were based on the main goal and set five research questions related to the given issue. Also, five hypotheses were set to answer whether CSR activities are considered a marketing tool in food companies (see the Section 3).

When formulating the research questions and hypotheses, we were based on previous knowledge from our research activities [43,44,45,46,47,48,49] and the research of recognized authors in the field of CSR [49,50,51,52,53,54,55,56,57,58].

#### 2.2.1. Research Questions

RQ1: What is the difference in the perception of CSR activities and the company’s good name between different age groups?

RQ2: Do respondents perceive CSR activities of food companies as a marketing tool?

RQ3: When purchasing food, do *respondents* make decisions based on whether a food company applies CSR?

RQ4: Is there a relationship between the respondents’ income and the maximum price difference they are willing to pay for socially responsible products?

RQ5: What percentage of the younger generation knows a concrete food company that applies CSR and is based in Slovakia?

#### 2.2.2. Hypotheses

**H1:**  *There is a difference between the perceptions of CSR activities as a good name of food companies between age categories*.

**H2:**  *More than 30% of respondents perceive the CSR activities of food companies as a marketing tool*.

**H3:**  *There are differences between different age categories and decision-making when buying food, based on whether the company is socially responsible*.

**H4**  : *There is a significant positive relationship between income and the maximum price difference that the respondent is willing to pay for a socially responsible product*.

**H5**  : *More than 50% of respondents aged 18–27 know a specific food company operating in Slovakia*.

Regarding Hypothesis 1, few research studies have been conducted focusing on the issue of CSR and its relation to the company’s good name which have declared that CSR initiatives would positively impact customers’ purchase intention and corporations’ brand [59,60]. When the customers find that the company cannot be trusted due to its unethical behavior, they might consider not purchasing its products [60] and will not perceive it as credible; i.e., they will not continue to spread its good name. Different initiatives would have various impacts on customers [61,62,63], and these impacts could be as positive as negative. Furthermore, different age categories and, thus, different generations can be assessed as a significant independent variable for the assessment of CSR perception; the attitude changes in the following way: though somewhat unexpected, the younger generation (1981–2000 year of birth) assess corporate social responsibility as less important compared to the older generation. While for those born between 1965 and 1980, the average importance of CSR is 4.35 on a 5-point scale, for the Y generation, it is a bit below 4, so quite a few youngsters are not considering the issue [64].

When formulating Hypotheses 2 and 5, we used the gained knowledge from our research activities [43,44,45,46,47,48,49] and the research of recognized authors in the field of CSR [49,50,51,52,53,54,55,56,57,58].

Currently, consumption trends profoundly changed [65] as many customers started to care about what they eat and moved towards the consumption of organic and healthy food [66,67], which could be regarded as a result of growing responsibility in the food industry [68]. Consumers are more environmentally conscious and take action to solve environmental problems by themselves [69]. The results obtained by Mercadé-Melé, Fandos-Herrera, and Velasco-Gómez [70] suggested that the main CSR activities carried out by companies in the agri-food sector are determining factors in consumers’ perceptions of the companies in terms of food safety, health, and food quality. Subsequently, these factors influence consumer satisfaction and loyalty in return. This knowledge was a base for Hypotheses 3.

When formulating Hypothesis 4, the practice shows that customers’ purchase intention would be reflected by various components, including willingness to pay more and recommend to others [71,72]. Thus, when corporations implement CSR initiatives aligned with the customers’ requirements, the customers would be willing to pay more and recommend it to others [73,74].

### 2.3. Statistical Analysis

The main focus of the research was to determine whether respondents are familiar with the concept of CSR of food companies and whether they know specific food companies based in Slovakia. We used various statistical methods to evaluate the data obtained from the questionnaire and the statistical verification of the hypotheses. We used frequency and contingency tables to calculate, summarize, and analyze the collected data from the questionnaire. Furthermore, we used statistical methods to verify hypotheses, such as the One sample ratio Z test, Pearson’s Chi-square, and Spearman’s rank correlation coefficient. The software IBM SPSS Statistics was used to process the statistical data.

## 3. Results

Currently, most food companies already act socially responsibly and show various activities; however, customers cannot connect with the concept of CSR or consider them only as a marketing strategy. As food companies try to build the company’s good name and brand image with these activities, the public can view these activities as untrustworthy. CSR activities in marketing communication can greatly benefit companies, increasing the sale of goods and services and creating competition in the food market.

### 3.1. Perception of CSR of Food Companies

The first hypothesis, which we statistically evaluate, focuses on the perception of the company’s CSR as a factor increasing the company’s good name depending on the age category. We hypothesize that age affects the perception of CSR (H1). The table below shows the respondents’ answers by age category. Out of the total number of 1254 respondents, 652 perceive CSR as a factor in increasing the company’s good name. This is 52% of the respondents. A total of 601 respondents (48%) do not perceive CSR as a factor that would increase the company’s good name. If the null hypothesis is valid, i.e., independence of CSR perception from age, we expect a similar ratio across all age categories.

The most numerous is the 18–27 years with 336 respondents (26.8%). A total of 192 (57.1%) perceive the company’s CSR as increasing its reputation. A total of 144 (42.9%) think the opposite. For individual age categories, this is the largest share of people who perceive CSR as contributing to the growth of the company’s good name. In the age groups 28–42 and 43–57 years, the differences are smaller and very similar to the overall ratio of answers (52% to 48%). The situation is different for the oldest group of 58 and more. Of 307 respondents in this age group, 143 perceive CSR as a factor in increasing the company’s good name (46.6%). More than half of the respondents, namely 164 (53.4%), do not consider CSR a factor in increasing the company’s good name.

The biggest differences are, as we expected, between the youngest and oldest groups. The Pearson Chi-Square test of independence will answer whether these differences are statistically significant, the results of which are shown in Table 1. The *p*-value of the Pearson Chi-Square test of independence is 0.066. We accept the null hypothesis. Age does not affect whether people perceive CSR as a factor that increases the company’s reputation.

### 3.2. Perception of CSR Activities as a Marketing Tool

The second hypothesis focuses on the perception of CSR activities of food companies. We assume that more than 30% of respondents perceive the CSR activities of food companies as a marketing tool (H2). Respondents had a choice of five options:

- 1-definitely not;
- 2-rather not;
- 3-I do not know;
- 4-rather yes;
- 5-definitely yes.

**Figure 2:**
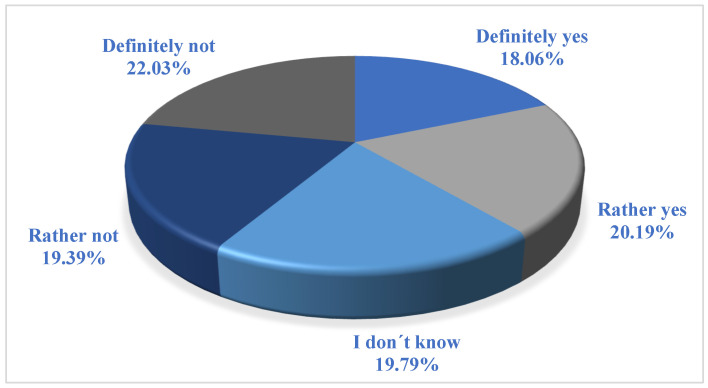
Perception of the CSR activities of food companies as a marketing tool. Source: Authors’ research and calculations, output IBM SPSS Statistics.

Figure 2 below shows the structure of the answers. The number of respondents who perceive CSR activities as a marketing tool includes respondents who answered rather yes or definitely yes. Of the 1253 respondents, 233 (19%) perceive CSR as a marketing tool. A total of 253 (20%) perceive CSR more as a marketing tool. A total of 486 respondents (39%) perceive CSR definitely or rather as a marketing tool.

The test statistic Z of the test is 6.78. The critical value at 95% confidence of a right-sided test is 1.65 (Table 2). We reject the null hypothesis. The share of respondents who perceive CSR activities as a marketing tool is statistically significantly higher than 30%.

### 3.3. Influence of CSR Activities on Decision-Making When Buying Food

Hypothesis 3 examines respondents’ decision-making when buying food concerning corporate social responsibility depending on age. We assume that age impacts people’s decisions when buying food.

Table 3 shows the respondents’ answers to the question, *“Do you decide when buying food based on whether the company is socially responsible?”.* Of 1253 respondents, 210 (16.8%) do not decide whether the company is socially responsible when buying food. A total of 251 (20%) respondents did not mention the possibility. A total of 259 people were neutral. A total of 533 respondents would rather decide (271–21.6%) or decide (262–20.9%) when buying food based on whether the company is socially responsible. The answers are close to 20%, except for definitely not (16.8%) and rather not (21.6%).

Divided by age categories, 23.8% (80) of respondents in the age group of 18–27 “certainly decide” and 23.8% (80) would “rather decide” to buy the food from a socially responsible company. In the age group 28–42 years, 19.2% (56) of respondents answered “certainly decide” and 19.2% (56) “rather decide,” and in the age group 43–57, answers were as follows: 24.8% (79) “certainly decide” and 17.3% (55) “rather decide.” The last age group of respondents (58 and more years) also proved that 18.2% (56) “certainly decide” and 23.1% (71) would “rather decide” to buy food from a socially responsible company.

The *p* value of the Chi-square test is 0.211 (Table 4). Therefore, we do not reject the null hypothesis. Deciding on food purchases concerning the company’s social responsibility is not influenced by age.

### 3.4. Willingness of Respondents to Buy a Socially Responsible Product

In the fourth hypothesis, we assume a significant dependence between income and the maximum price difference the respondent is willing to pay for a socially responsible product. We assume that the willingness to pay for a socially responsible product increases with income. Both variables were converted to an ordinal scale (Table 5).

We used Spearman’s rank correlation coefficient (Table 6) to measure the relationship between variables, which acquired a value of −0.008 with a *p*-value of 0.766. There is no significant relationship between the variables, confirmed by the coefficient value close to zero.

### 3.5. Awareness of Food Companies in Slovakia

**Figure 3:**
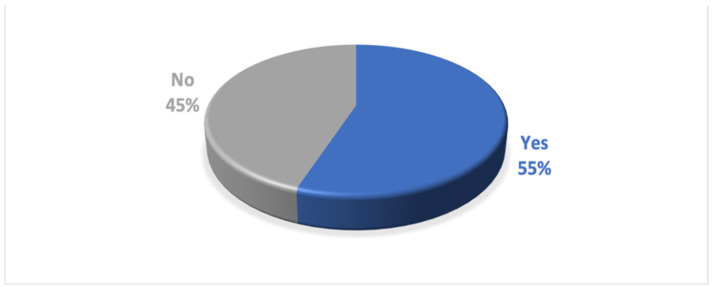
Knowledge of a specific food company operating in Slovakia that is socially responsible. Source: Authors’ research and calculations, output IBM SPSS Statistics.

The fifth hypothesis refers to the question, *“Do you know a food company operating in Slovakia that is socially responsible?”* We want to verify the hypothesis that more than 50% of respondents in the age category 18–27 know a socially responsible food company. Out of 336 respondents aged 18–27, 186 know a socially responsible food company operating in Slovakia, which means 55 % of the respondents. A total of 150 respondents (45%) do not know such a food company (Figure 3).

The value of the Z test statistic is 1.96 (Table 7). The test statistic is higher than the critical value (1.65). We reject the null hypothesis of equality of shares. The share of people aged 18–27 who know a socially responsible food company operating in Slovakia is more than 50%.

## 4. Discussion

Vaaland et al. [75] opened a discussion regarding the status of the theory of corporate social responsibility (CSR) applied in the marketing context and asked whether to what extent and how the discipline of marketing has addressed CSR. Our research confirmed that one could see the generation bias in personal CSR perception, which is in line with existing literature (for instance, Haski-Leventhal et al. [76] and Rosati et al. [77] had found the same tendency worldwide and in Italy, accordingly). The same was true for our sample; on the other hand, our research had gone further to evaluate the moderating role of age in CSR perception, but this part of our Hypothesis 2 was not supported. As suggested by other researchers, age is a factor that influences CSR perception directly. However, we did not find support for our suggestion that age can also be a moderating factor.

The research also revealed that aging is linked to increased cynicism and distrust, which may also be applied to the perception of CSR activities [78]. Our results support the previous findings of other studies, which have demonstrated that age predicts CSR knowledge and customers’ attitudes and behavior [79]. As it has been proven by a few researchers [79,80,81,82], younger customers are more concerned about CSR than older customers and make a greater effort to act accordingly. For example, Arlow [79] believes that age plays the most important role in customer orientation towards CSR, with the youngest people showing the greatest interest in CSR. Similarly, Anderson and Cunningham [80] show that both younger customers and managers present a clearer CSR orientation, as well as the mentioned results are confirmed by Aldag and Jackson [81], who contrast the inverse relationship between age and CSR orientation: older customers are less interested in CSR than younger customers. We were to assess the influence of age (generation) on the personal perception of CSR, which formed Hypothesis 1: Age influences respondents’ perception of corporate social responsibility. Based on the obtained data, this hypothesis was partly supported by the previous research of [64], which declared that age appears to be an independent variable but not a moderator.

The stakeholders care whether a company follows a socially responsible path [83]. Stakeholders expect companies to communicate their CSR efforts to the audience [32], called CSR advertisement [84]. According to McWilliams et al. [85], CSR practices are strategic and apply to marketing actions [11] regarding building, e.g., loyalty, brand reputation, or brand equity. Some authors argue that none of the CSR practices analyzed significantly influence corporate reputation [83]. Fombrun and Shanley [86] concluded that investing in CSR may be an important element of product differentiation and reputation building.

Food no longer has a mere nutritional function but becomes a means of satisfying personal pleasure [87]. According to many researchers, a positive relationship exists between companies with implemented CSR practices and consumer attitudes towards these companies and their products [88,89].

Only some customers care about CSR as a buying criterion because most customers care about price, quality, and convenience, which prevail due to the insufficient knowledge of CSR [90]. However, other authors argue that consumers at least care about environmental concerns, which is an important factor in consumer choice and decision-making [82,91]. According to the literature review, we can assume that consumer demand is linked to business activity’s ethical dimension, as Boccia et al. stated [87]. However, CSR is not a criterion of choice at the time of purchase, as traditional selection criteria prevail, mainly brand and price [87]. Research by Saldivar and Zolfagharian [92] was aimed at the Expectancy Confirmation/Disconfirmation (ECD) paradigm to extend the knowledge about consumer response to CSR. While several scholars have identified consumer satisfaction with the firm as a salient variable that justifies investment in CSR, few have considered consumers’ CSR perceptions against the backdrop of their CSR expectations, and none have done so empirically. Their research examined whether ECD, which incorporates perceptions and expectations regarding CSR, affects satisfaction, referral, and willingness to pay a premium. The research results by Bryła [93] confirmed the relationship between the positive perception of European quality signs and the willingness to pay a higher price for origin food, which could also be one element of CSR.

There are examples of the relationship between CSR, the company’s image, and customer attitudes in the academic area [94]. According to some studies [95,96,97,98,99,100], CSR can multiply the positive image brand value and provide a solid base for competitive advantages. Results of the study conducted by Harjoto et al. [101] proved that CSR strengthens the image of the company and the brand’s value when CSR irresponsible activities lower the brand’s reputation.

Because of the impact of rationality and irrationality on consumer behavior, a company that behaves socially responsibly can upgrade its value in the customer’s mind and increase its image in the market [102,103], which aims for customer satisfaction and purchase the products or services offered by this company as the customer trusts the company.

This is why it is essential to communicate the company’s CSR activities to the public and why it can be assumed that companies are aware of this and act accordingly.

## 5. Conclusions

The public expects food companies to show CSR activities in all three pillars of CSR—people, planet, and profit. On the threshold of the 3rd millennium, CSR is strongly connected with rationality and irrationality in creating preferences in consumer shopping behavior. Fulfilling the requirements and ideas of customers is important for the company to be able to present the given activities so that they appear trustworthy correctly.

Trustworthiness is one of the key factors in this direction for customers and their consumer behavior, which the CSR activities of food companies can influence.

Based on the chosen issue, we conducted a questionnaire survey focused on CSR activities as a marketing tool in the food market, aiming at CSR activities and marketing communication in the conditions of food businesses in Slovakia. Any other researcher in Slovakia still needs to carry out research with such a focus.

The survey results with 1254 participating respondents showed that, although they are familiar with the concept of CSR, a large percentage of respondents either need to learn these activities or pay more attention to them. This may be because some CSR activities need to be actively applied and communicated by food companies.

Based on our conducted research, customers perceive the CSR activities of food companies in most cases as a marketing communication tool that builds the company’s good name and image.

However, our research also has some limitations. We focused on respondents from Slovakia and the perception of food companies’ CSR activities in the Slovak Republic’s territory. We are also aware that CSR is rapidly developing, and the issue described in the submitted contribution may continue to develop and change. Based on this, new possibilities and trends for future research will be developed. In the future, the solved issue can be investigated from the point of view of respondents in other E.U. countries and food companies of selected countries, which can be an interesting basis for comparing selected countries and the use of CSR activities as a marketing tool on the food market not only in Slovakia but also abroad.

The submitted contribution creates a solid basis for further research and practical application in using CSR activities in Slovakia and E.U. countries on the food market.

## Figures and Tables

**Table 1:** Evaluation of H1: There is a difference between the perceptions of CSR activities as a good name of food companies between age categories.

			Do you Perceive the Company’s CSR as a Factor in Increasing the Company’s Good Name?		Total
			Yes	No	
Age group of respondents	18–27 years	Count	192	144	336
		% within Age	57.1%	42.9%	100.0%
		% within Do you perceive the company’s CSR as a factor increasing the company’s good name?	29.4%	24.0%	26.8%
	28–42 years	Count	151	141	292
		% within Age	51.7%	48.3%	100.0%
		% within Do you perceive the company’s CSR as a factor increasing the company’s good name?	23.2%	23.5%	23.3%
	43–57 years	Count	166	152	318
		% within Age	52.2%	47.8%	100.0%
		% within Do you perceive the company’s CSR as a factor increasing the company’s good name?	25.5%	25.3%	25.4%
	58 years and more	Count	143	164	307
		% within Age	46.6%	53.4%	100.0%
		% within Do you perceive the company’s CSR as a factor increasing the company’s good name?	21.9%	27.3%	24%
Total (all age groups of respondents)		Count	652	601	1253
		% within Age	52.0%	48.0%	100.0%
		% within Do you perceive the company’s CSR as a factor increasing the company’s good name?	100.0%	100.0%	100.0%

Source: Authors’ research and calculations, output IBM SPSS Statistics.

**Table 2:** Evaluation of H2: More than 30% of respondents perceive CSR activities of food companies as a marketing tool.

*n* (Number of Respondents)	1253
x (number of respondents who perceive CSR as a marketing tool)	486
p (share of respondents who perceive CSR as a marketing tool)	0.3879
p_0_ (expected share)	0.3
Z (Test statistic of one sample proportion test)	6.7874
Critical value (alfa = 0.05, right-sided test)	1.6449
*p*-value	0.000

Source: Authors’ research and calculations, output IBM SPSS Statistics.

**Table 3:** Evaluation of H2: More than 30% of respondents perceive CSR activities of food companies as a marketing tool.

			When Buying Food, Do You Decide Whether the Company Is Socially Responsible? (1-Certainly Not; 2-Rather Not; 3-Do not Know; 4-Rather Yes; 5-Certainly Yes)					Total
			1	2	3	4	5	
Age group of respondents	18–27 years	Count	56	63	57	80	80	336
		% within Age	16.7%	18.8%	17.0%	23.8%	23.8%	100.0%
	28–42 years	Count	47	59	74	56	56	292
		% within Age	16.1%	20.2%	25.3%	19.2%	19.2%	100.0%
	43–57 years	Count	52	64	68	79	55	318
		% within Age	16.4%	20.1%	21.4%	24.8%	17.3%	100.0%
	58 years and more	Count	55	65	60	56	71	307
		% within Age	17.9%	21.2%	19.5%	18.2%	23.1%	100.0%
	Total	Count	210	251	259	271	262	1253
		% within Age	16.8%	20.0%	20.7%	21.6%	20.9%	100.0%

Source: Authors’ research and calculations, output IBM SPSS Statistics.

**Table 4:** Results of the Chi-Square Tests.

Chi-Square Tests			
	Value	df	Asymptotic Significance (2-Sided)
Pearson Chi-Square	15.594 ^a^	12	0.211
Likelihood Ratio	15.619	12	0.209
N of Valid Cases	1253		

^a^ 0 cells (0.0%) have an expected count of less than 5. The minimum expected count is 48.94. Source: Authors’ research and calculations, output IBM SPSS Statistics.

**Table 5:** Ordinal Scale.

What Is Your Average Disposable Income?	What Maximum Price Difference Would Be Acceptable for You to Prefer Such a Product?	Coding
Less than 500 EUR	none	1
501–1000 EUR	below 10%	2
1001–1500 EUR	10–19.9%	3
1501–2000 EUR	20–30%	4
More than 2000 EUR	over 30%	5

Source: Authors’ research and calculations, output IBM SPSS Statistics.

**Table 6:** Results of Spearman’s rank correlation coefficient.

Correlations			
			What Maximum Price Difference Would Be Acceptable for You to Prefer Such a Product?
Spearman’s rho	What is your average disposable income?	Correlation Coefficient	−0.008
		Sig. (2-tailed)	0.766
		N	1253

Source: Authors’ research and calculations, output IBM SPSS Statistics.

**Table 7:** Evaluation of H4: More than 50% of respondents aged 18–27 know a specific food company operating in Slovakia.

*n* (Number of Respondents in Age 18–27)	336
x (number of respondents who know socially responsible food companies in Slovakia)	186
p (share of respondents who know socially responsible food companies in Slovakia)	0.5536
p_0_ (expected share)	0.5
Z (Test statistic of one sample proportion test)	1.9639
Critical value (alfa = 0.05, right-sided test)	1.6449
*p*-value	0.0248

Source: Authors’ research and calculations, output IBM SPSS Statistics.

## Data Availability

The data used to support the findings of this study can be made available by the corresponding author upon request.
